# Spilling the beans on duplicated genes: unraveling the mechanisms underlying transcriptional divergence in soybean

**DOI:** 10.1093/plcell/koaf283

**Published:** 2025-12-17

**Authors:** Róisín Fattorini

**Affiliations:** Assistant Features Editor, The Plant Cell, American Society of Plant Biologists; Institute of Molecular Plant Sciences, University of Edinburgh, Edinburgh EH9 3BF, United Kingdom

Gene duplication is of great evolutionary importance because it gives rise to new genes (Des Marais and Rausher 2008). By reducing the selective constraints imposed on one or both gene copies, gene duplication enables the acquisition of new functions that contribute toward evolutionary novelty and ultimately adaptation (Arsovski et al. 2015). Duplicated genes arise from either whole-genome duplications that affect all the nuclear-encoded genes or small-scale duplications that are restricted to small genomic regions. Duplicated genes often acquire divergent spatiotemporal expression patterns or variation in transcriptional levels that stem from differences in how *cis*-regulatory elements are used (Zhang et al. 2025). Key evolutionary insights can be gained from investigating gene expression dynamics and the regulatory processes underlying them in the context of gene pair derivation.

In new work in *The Plant Cell*, Xiang Li and colleagues (Li et al. 2025) investigated the dynamics of gene duplication in soybean (*Glycine max*), focusing on transcription and *cis*-regulatory processes. The authors used previously published datasets of single-nucleus RNA sequencing and single-cell transposase-accessible chromatin sequencing (Zhang et al. 2025) to explore gene expression and the *cis*-regulatory landscape with single-cell resolution. The expression of duplicated genes was assessed across cell types within 7 tissues: root, nodule, hypocotyl, and seed at the globular, heart, cotyledon, and early-maturation stages. The accessible chromatin regions (ACRs) associated with duplicated genes were also analyzed, as most transcription factors bind to *cis*-regulatory elements in these regions to regulate gene expression (Zhang et al. 2025). Expression divergence between duplicated genes within tissues was largely shaped by genetic variation in ACRs. When duplicated gene pairs were grouped by within-tissue expression pattern dynamics, the majority were found to have a single gene expressed, with the second gene inactive in a given tissue (mono-expression). Across-tissue differences in expression patterns were more likely to be attributed to dynamic changes in ACR chromatin accessibility, as the underlying genetic variation in ACRs between duplicates is constant across tissues. When examined in the context of duplication mechanisms, expression patterns were found to be similar in gene pairs originating from whole-genome duplication and those from dispersed duplication (a form of small-scale duplication where duplicate gene copies are scattered across the genome). Genes derived from the remaining small-scale duplication mechanisms (eg tandem duplication), however, had more similarity in expression patterns (Fig. 1).

**Figure 1:**
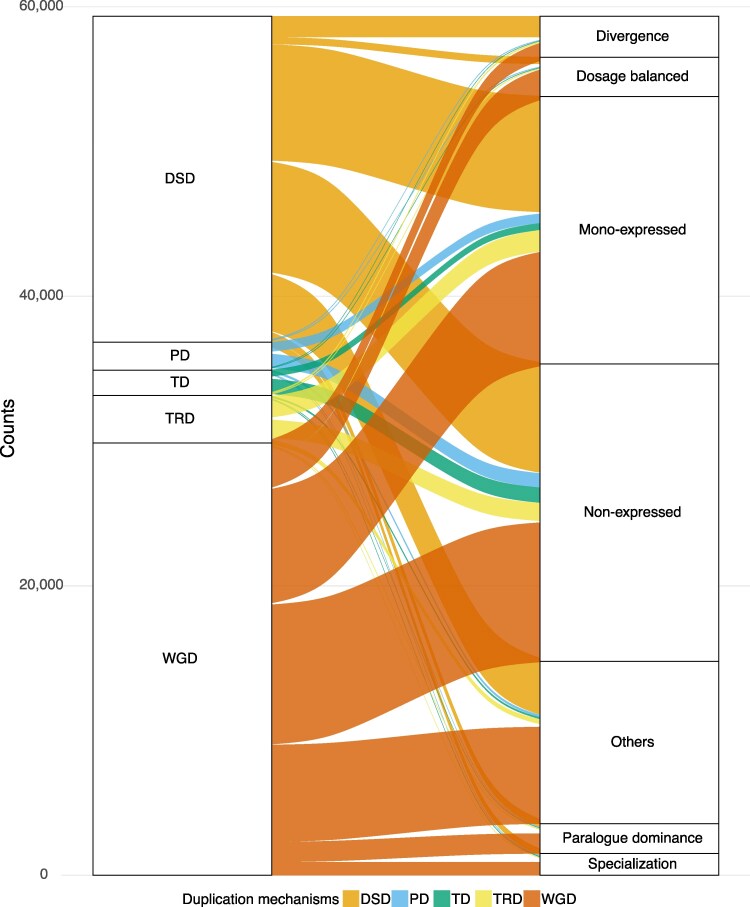
Schematic of gene expression patterns arising from gene duplication. Genes are categorized based on duplication mechanism (left panel): dispersed duplication (DSD), proximal duplication (PD), tandem duplication (TD), transposed duplication (TRD), and whole-genome duplication (WGD). Gene expression patterns are listed (right panel). The relative frequency of each transition is represented by flow width, illustrating how different duplication mechanisms contribute to expression variation within tissues. Reprinted from Li et al. (2025), Figure 2.

Soybean has undergone 2 rounds of whole-genome duplication: one ancestral and one more recent. Gene sets consisting of 4 whole-genome duplication–derived gene copies were used to investigate the evolution of *cis*-regulatory regions. By integrating expression dynamics, the ACRs likely responsible for cell type–specific expression could be identified through analyses of regulatory sequence conservation and chromatin accessibility profiles. For example, in one gene set, one gene pair exhibited nonspecific expression, whereas the other gene pair was specifically expressed in the seed-coat epidermis. Three ACRs were found to have greater chromatin accessibility in seed-coat epidermal cells, and 2 of them were associated with the specifically expressed genes, likely contributing toward the expression difference among duplicated genes. The cellular resolution acquired from the single-cell datasets, assessed in a gene evolutionary context, enabled precise inferences about the evolution of these regulatory elements contributing to cell type–specific expression.

Ultimately, this research contributes to our evolutionary understanding of gene paralogs. The use of single-cell datasets allowed analyses of transcriptional and regulatory divergence both within and across tissues. Li et al. (2025) provide a valuable exploration of the mechanisms driving gene divergence following duplication events in an important crop species.

## Recent related articles in *The Plant Cell*

Wang et al. (2021) created high-resolution Hi-C maps for soybean and common bean to investigate 3D chromosome structure and the contribution of dynamic chromatin structures to gene expression. They found that expression bias of genes derived from whole-genome duplication in soybean may be due, in part, to divergence of chromatin interactions.

Almeida-Silva and Van de Peer (2025) used spatial transcriptomic data sets from 5 angiosperm species to investigate gene expression evolution after gene duplication events. Genes derived from different duplication mechanisms had different expression levels and spatial expression profiles.

## Data Availability

No new data were generated or analyzed in support of this research.

## Dive Curated Terms

The following phenotypic, genotypic, and functional terms are of significance to the work described in this paper:
